# Seasonal weight changes in laboratory ferrets

**DOI:** 10.1371/journal.pone.0232733

**Published:** 2020-08-07

**Authors:** Eleanor J. Jones, Katarina C. Poole, Joseph Sollini, Stephen M. Town, Jennifer K. Bizley

**Affiliations:** The Ear Institute, University College London, London, England, United Kingdom; University of Texas Southwestern Medical Center, UNITED STATES

## Abstract

Ferrets (*Mustela putorius furo*) are a valuable animal model used in biomedical research. Like many animals, ferrets undergo significant variation in body weight seasonally, affected by photoperiod, and these variations complicate the use weight as an indicator of health status. To overcome this requires a better understanding of these seasonal weight changes. We provide a normative weight data set for the female ferret accounting for seasonal changes, and also investigate the effect of fluid regulation on weight change. Female ferrets (n = 39) underwent behavioural testing from May 2017 to August 2019 and were weighed daily, while housed in an animal care facility with controlled light exposure. In the winter (October to March), animals experienced 10 hours of light and 14 hours of dark, while in summer (March to October), this contingency was reversed. Individual animals varied in their body weight from approximately 700 to 1200 g. However, weights fluctuated with light cycle, with animals losing weight in summer, and gaining weight in winter such that they fluctuated between approximately 80% and 120% of their long-term average. Ferrets were weighed as part of their health assessment while experiencing water regulation for behavioural training. Water regulation superimposed additional weight changes on these seasonal fluctuations, with weight loss during the 5-day water regulation period being greater in summer than winter. Analysing the data with a Generalised Linear Model confirmed that the percentage decrease in weight per week was relatively constant throughout the summer months, while the percentage increase in body weight per week in winter decreased through the season. Finally, we noted that the timing of oestrus was reliably triggered by the increase in day length in spring. These data establish a normative benchmark for seasonal weight variation in female ferrets that can be incorporated into the health assessment of an animal’s condition.

## Introduction

Domesticated ferrets (*Mustela putorius furo*) are valuable animal models for a wide range of biomedical research areas, including: neuroscience [1–6], drug development [7] and respiratory diseases such as Influenza and Severe Acute Respiratory Syndrome (SARS) [8,9] including the new coronavirus strain, SARS-CoV-2 [10]. In laboratory animals exposed to scientific procedures, a standard approach to monitoring health status is to measure body weight. Weight loss is a key indicator of health problems, and therefore understanding the factors that contribute to natural variation in body weight is critical for correctly monitoring an animal’s condition. Ferrets undergo significant variation in their body weight seasonally; however, there are currently no normative data available to provide a benchmark for the expected seasonal weight changes. Seasonal variations may mask or exaggerate changes in body weight due to an experimental procedure or change in health status and thus must be integrated into assessments of a ferret’s health status.

Seasonal weight changes have been demonstrated in multiple species independent of diurnality, including monkeys [11,12], raccoons [13], hamsters [14] and rodents [15]. There are a range of potential factors that elicit seasonal weight changes, but temperature and day length are key triggers, which are ultimately crucial for survival [16].

Ferrets are members of the mustelid family and have been domesticated from European polecats, a species which was native to western Euroasia. Seasonal weight changes have been observed in polecats and other closely related species such as mink. These weight changes are seen as adaptations to the differing energy intake and expenditure requirements of winter and summer [17,18]. In animal care facilities, daylight hours can be easily regulated and are often set at a 12-hour light cycle (12-hours ON, 12-hours OFF) or synchronised with the external environment; for example, varying from a minimum 8-hour cycle in winter (8-hours ON, 16-hours OFF) to maximum 16-hour cycle in summer (16-hours ON, 8-hours OFF) [19–22]. Variation in the photoperiod can change factors such as eating habits, coat thickness, sleep and activity levels—all of which may contribute to normal and possible abnormal weight changes. Previous research has demonstrated that ferret weights increase as hours of daylight decrease, leading to sinusoidal weight fluctuations with annual light cycle [23,24]. Contrastingly, in another study where the sleep habits of two male ferrets were tracked, light/dark schedule was shown to have no effect on their weight [25].

In addition to body weight, changes in photoperiod have also been linked to the timing of the oestrus cycle, which occurs once per year in female ferrets [21,23,26,27]. One of the first studies showed that sexual activity in ferrets increased when light duration or intensity increased [28]. Since then, further research has described ferret oestrus as seasonal and photoperiod activated [29]. The relationship between photoperiod, oestrus and body weight is unknown, but Donovan (1986) concluded that while there was not a critical weight to trigger oestrus, oestrus does require a minimum weight of around 420g.

The aim of this study is to provide data on the normative weights of female ferrets, accounting for seasonal changes over multiple years. In addition, we document changes in weight that occur due to water regulation. We hypothesized that controlled light exposure in animal care facilities would induce naturalistic fluctuations in the ferrets’ body weight.

## Methods

### Ethics statement

All the animals in this study were maintained for the purpose of investigating the neural basis of hearing, undergoing experimental procedures that were approved by local ethical review committees (Animal Welfare and Ethical Review Board) at University College London and The Royal Veterinary College, University of London and performed under license from the UK Home Office (Project License 70/8987) and in accordance with the Animals (Scientific Procedures) Act 1986.

### Animals

The data from 39 healthy female pigmented ferrets (0.5–4 years on study completion) were used for this study. All animals underwent behavioural testing in psychoacoustic tasks which continued after the completion of this study. These tasks required regulated access to water. Water was available during twice-daily testing sessions, with supplementary wet food and/or water provided to ensure animals received a minimum of 60 ml/kg of water (estimate based on daily measurements made on ferrets with access to free water in the animal facility at the University of Oxford[30]). Testing took place from Monday to Friday typically in a three-weeks on, and one week off, schedule. This ensured that ferrets did not experience water regulation more than 50% of the time. When not participating in behavioural testing, animals had free access to water. During testing periods, each animal was weighed daily using digital scales (Salter, UK) prior to their morning testing session. Data were obtained from all available animals between May 2017 and August 2019 (months of participation, mean± SD: 11 months ±4.1).

Animals were housed at 15–24°C in social groups (n = 2 to 8 ferrets) and had free access to high-protein food pellets. Animals lived in enriched cages and freely exercised during daily cage cleans, with the opportunity to interact with humans, other ferrets and a variety of enrichment facilities (e.g. tunnels and balls). Our colony was comprised exclusively of female ferrets and typically contained between 25 and 30 animals.

**Fig 1:**
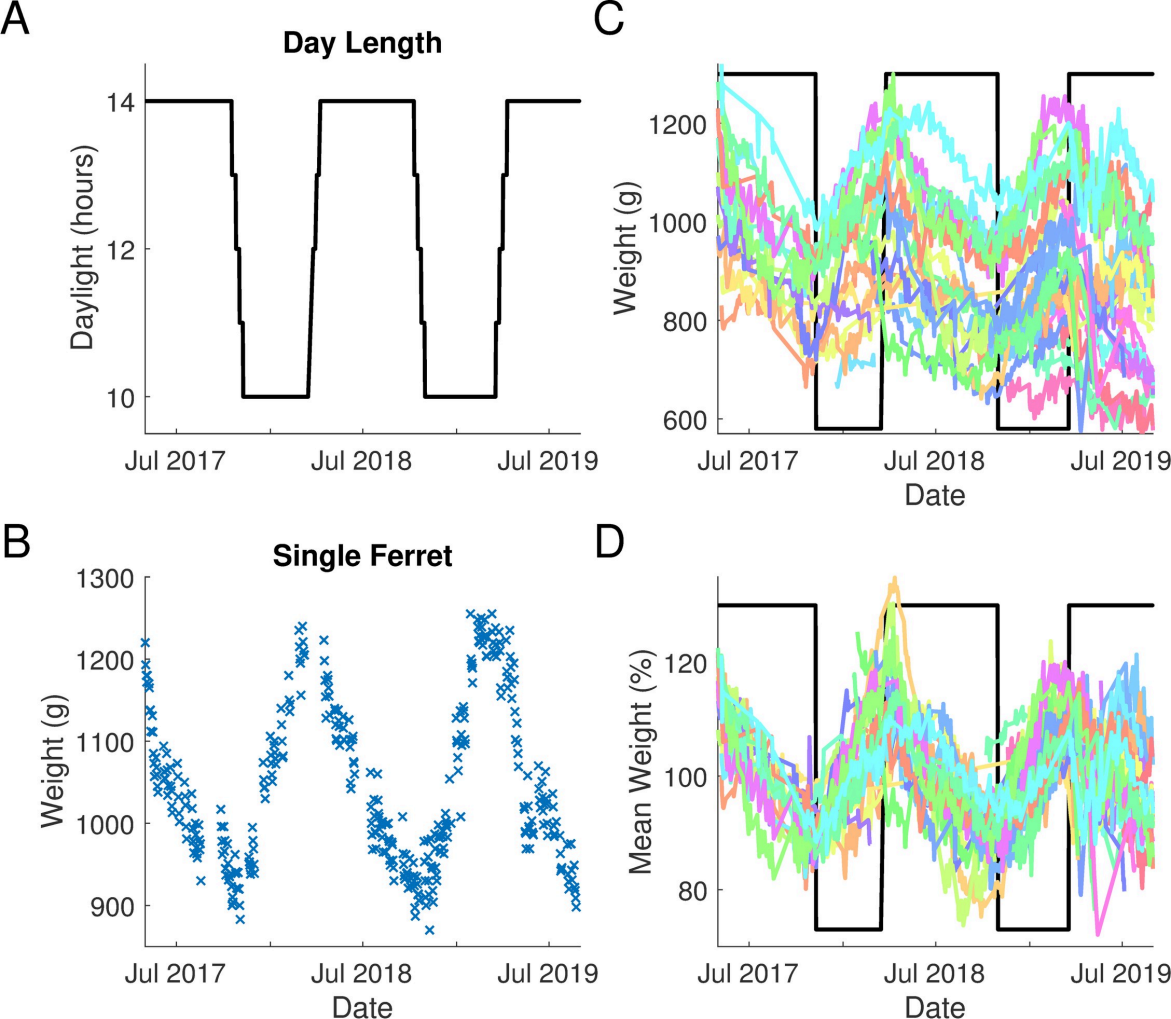
Seasonal fluctuations in body weight. **A.** Relative light hours and clock change transition periods ferrets were exposed to during the 28-month period. **B.** Weight change for a single ferret (F1606) between May 2017 and August 2019. **C.** Absolute weight of all ferrets (n = 39) between May 2017 and August 2019. **D.** Seasonal variation in weight expressed as a percentage change from each ferret’s average bodyweight.

The light cycle was changed in accordance with UK daylight savings: during ‘winter’ (October to March) ferrets were exposed to 10 hours of light and 14 hours of dark; during ‘summer’ (March to October) this was reversed to 14 hours of light and 10 hours of dark. The animal facility in which the animals were housed was windowless, and thus animals did not have access to natural light. The transition between ‘seasons’ was staggered such that timings were changed one hour per week over 4 weeks, centred on clock change for UK daylight saving time (Fig 1A).

The age of each ferret was calculated from the approximate date of birth provided by the supplier (Highgate Farms, UK). Also available for each animal was the oestrus time, which was estimated from the record of each animal’s yearly hormone injection (0.5ml s.c. Proligestone, Delvosteron, Intervet). Hormone injections were given within 24–72 hours of animals exhibiting visible signs of oestrus, in order to suppress oestrus until the following spring and thus prevent life-threatening anaemia experienced by females in sustained oestrus [31].

### Data analysis

Data were recorded and analysed in Matlab (version R2018a, MathWorks Inc, MA, USA) using custom written scripts. Weight measurements were either examined in absolute terms or relative to each animal’s long-term average (calculated from all available data across both seasons). To examine day-light triggered weight changes, data from summer and winter were considered independently in terms of weeks from the transition to shorter/longer days. To assess whether various factors (mean body weight, number of weeks since the change in light cycle and whether the ferret had been on water regulation the previous week) influenced the rate of weight loss/gain we calculated the change in weight in each week (from measurements made on a Monday morning) calculating the % change between week *i* and week *i+1*, relative to the weight recorded in week *i*.

ANOVAs were performed in SPSS (IBM) using Greenhouse-Geisser corrections for violations of sphericity where appropriate.

## Results

The weights of 39 female ferrets were recorded as part of their daily health monitoring through multiple cycles of summer and winter light cycles (Fig 1A). Over this period and across animals, weight values ranged from 553g-1350g. There was considerable variation both across animals, with average weights spanning 693 to 1195g (population mean ±SD; 864.8 ±119.0g) and also within each animal across seasons. For example, the animal shown in Fig 1B weighed 1240g on 5^th^ March 2018, and 870g on the 9^th^ of November that year, a change of 370g over nearly 7 months. The average standard deviation across all measurements was 64.9 (±30.3g), or equivalently, 7.45% ±3.25% of each animals’ mean weight. Here, we sought to determine how variation in body weight was linked to seasonal changes and fluid regulation during behavioural testing.

### Female ferrets show significant seasonal weight variations

When weights are considered over time, cycles emerge that correlate with the seasonal light changes (Fig 1). The pattern of weight change for one ferret over the collection period is shown in Fig 1B. All ferrets conformed to a similar seasonal pattern of weight change with weight greatest in April (when lights were altered to their summer day length) and lowest in October (when the light cycle was switched to winter day lengths, Fig 1C and 1D). The observed decreases in weight during the summer period and increases in weight over the winter months resulted in approximately sinusoidal weight fluctuations over the two-year measurement period.

**Fig 2:**
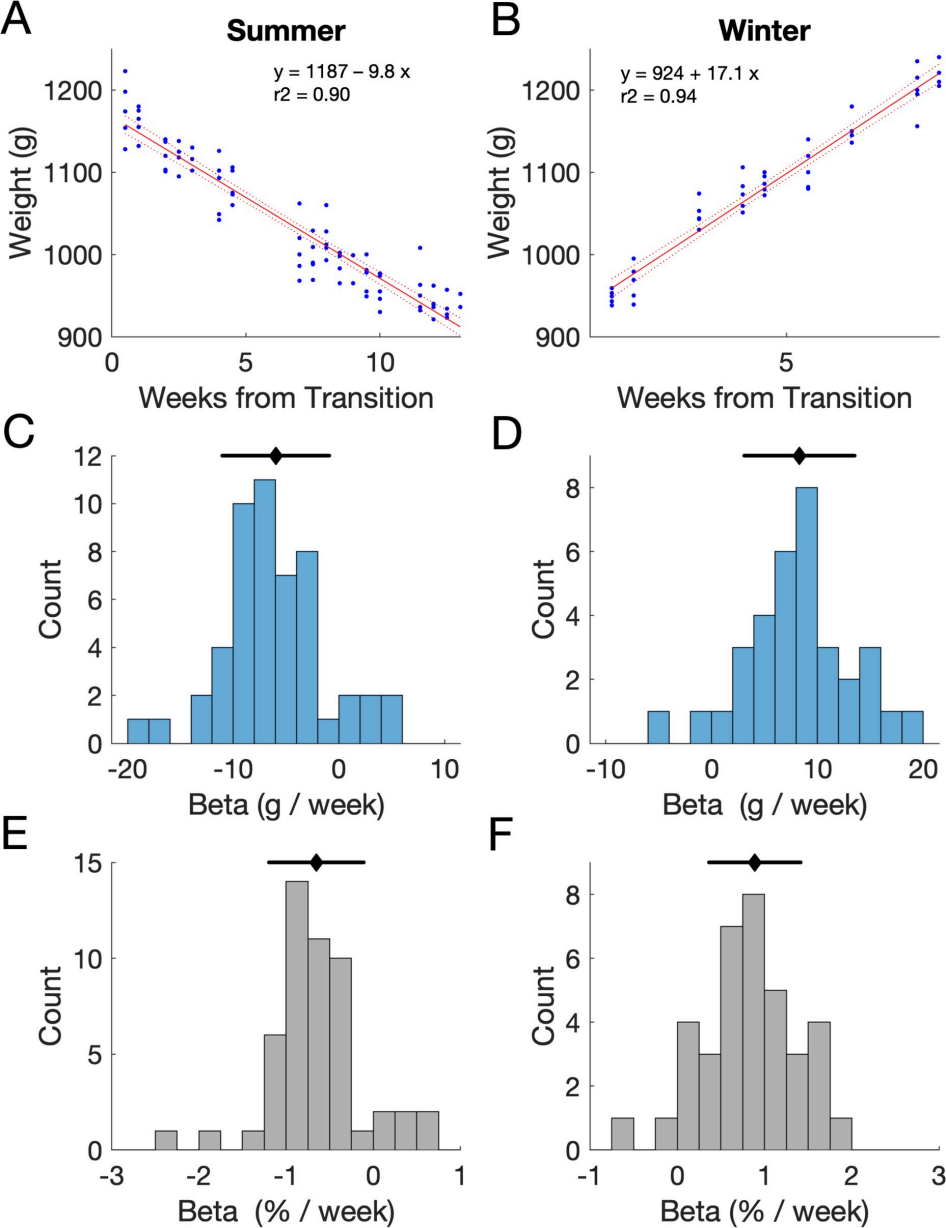
Seasonal weight changes. **A-B** Representative data from one animal (F1606) during summer 2018 **(A)** and winter 2017/18 **(B)**. Symbols indicate individual weight measurements; plotted according to the number of weeks since the transition to summer light cycles. Line indicates the regression fit (and confidence bounds). Regression coefficients, Summer: β = -9.84 g/week or -0.94% per week (t = -26.8, p<0.001); Winter: β = 17.01 g/week or 1.66% per week (t = 27.8, p<0.001). **C-D** Regression coefficients for all unique animal-transition combinations between 2017–2019 during summer (**C**, n = 51 ferret x transition combinations, 33 unique animals) and winter (**D**, n = 37 ferret x transition combinations, 28 unique animals). Black lines indicate the mean and standard deviation. **E-F** Regression coefficients from C-D expressed as a percentage of long-term mean body weight.

To quantify the observed changes in weight with season, we divided weight measurements into ‘summer’ and ‘winter’ periods according to day length (summer = 14 hours daylight, winter = 8 hours daylight), considering time as the number of weeks since the transition to longer/shorter days. For each animal and season in which we had at least 8 weeks of data, we performed a linear regression to determine the relationship between time (in weeks) and body weight (Fig 2A and 2B). In summer, there was a statistically significant relationship between time and body weight during the summer period for all animals (33 unique animals, some measured across multiple summers yielding 51 animal x season combinations; R^2^ (mean; min to max) = 0.59; 0.07 to 0.96, p < 0.05 (49/51 p<0.001). In winter, there was a significant relationship between week and body weight for 33/37 animal-transitions (28 unique animals; R^2^ (mean; min to max), 0.74; 0.10 to 0.97 p < 0.05, 31/33 p<0.001). We used the resulting regression coefficients (β) to determine the predicted weight change per week. We expressed weights in grams (Fig 2C and 2D) and also relative to their starting weight (Fig 2E and 2F). Measuring weight changes in this way allowed us to see a stereotyped pattern of weight loss/gain across animals. Weight changes were negative in summer (-6.0 g/week ±5.1 g/week; -0.65% ±0.55%) and positive in winter (+8.3 g/week ± 5.2 g/week; +0.89% ±0.53%), consistent with the pattern of weight changes observed across the year.

### Water regulation is associated with predictable weight variation

We next consider the impact of fluid regulation on body weight. The ferret is a popular model for neuroscience research as animals can be readily trained in a variety of complex behavioural tasks using water as a positive reward [32–34]. The animals that formed this dataset performed psychoacoustic tasks and were weighed as part of their daily health monitoring while on water regulation. Water regulation typically took place over a 5-day cycle, with water being removed from the home cage from Sunday night until Friday afternoon. We sought to quantify the impact of regulation on body weight, and whether there was any interaction with the seasonal changes reported above.

**Fig 3:**
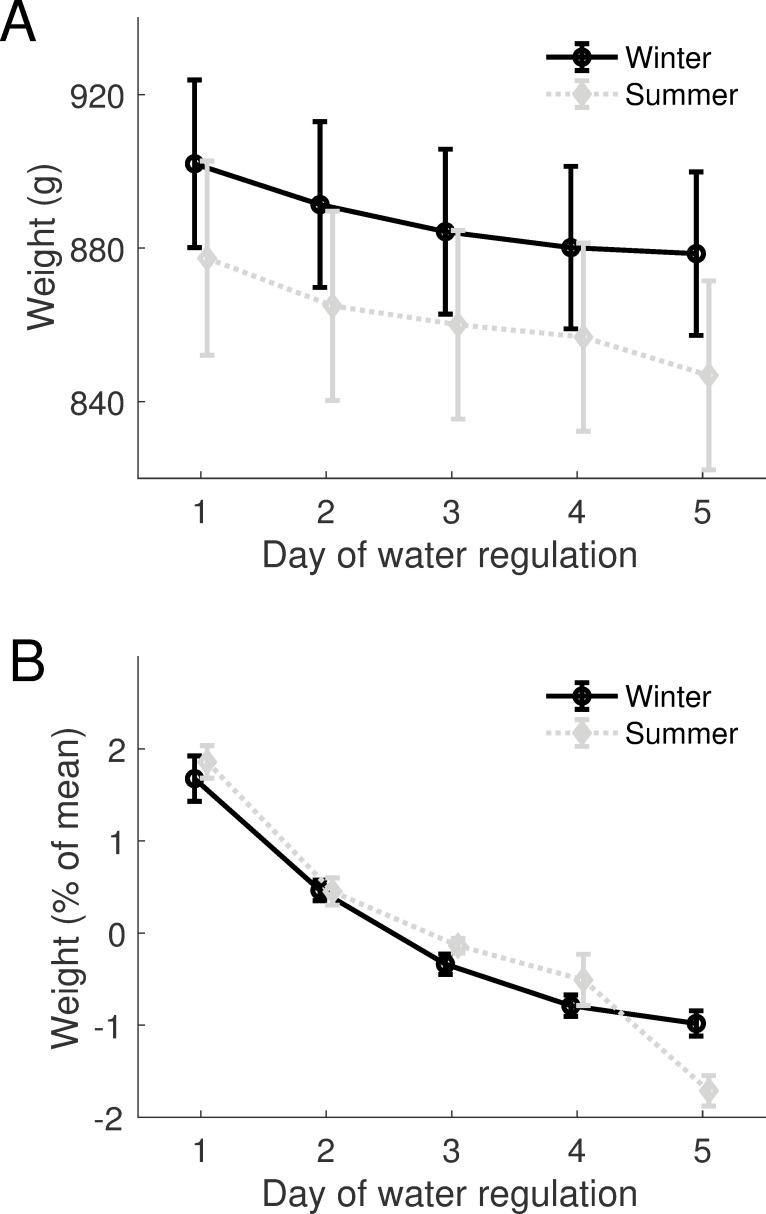
Weight changes resulting from water regulation. **A-B** Water regulation related weight changes for 30 animals in which both summer and winter data were available. **A** mean± SEM weight in grams for each day of water regulation for summer and winter periods. **B** mean ± SEM weight change expressed as a percentage change from the weight measurement made on a Monday morning (day 1).

We divided data into summer and winter periods and compared the weight across the 5 days of water regulation in absolute terms (Fig 3A) or relative to each animal’s long-term mean summer or winter weight (Fig 3B). Both metrics showed that weight declined across days on water regulation, although the trends differed between summer and winter. In the winter, weight loss plateaued at the end of the week, whereas in the summer weight loss appeared to continue more linearly through the week. These results were confirmed statistically using a two-way repeated measures ANOVA to analyse absolute body weight, where we found a main effect of day (F_4, 116_ = 59.4, p<0.001) and interaction between day and season (F_4, 116_ = 3.51, p = 0.023). Similar results were also found when analysing relative body weight, where again both main effect of day (F_4, 116_ = 59.1, p < 0.001) and interaction between day and season (F_4, 116_ = 3.93, p = 0.014) were significant. In neither analysis was there a significant main effect of season (absolute weight F_1,29_ = 3.662, p = 0.066; relative; F_1,29_ = 0.29, p = 0.593).

### Additional considerations for estimating body weight change

Linear regression indicated a significant relationship between time and body weight in both summer and winter (Fig 2). We next used a GLM based approach to further understand what factors influenced the rate at which animals lost / gained weight through the course of a season. Firstly, we asked whether the animals starting weight influenced the rate of weight loss (i.e. did larger animals lose / gain a larger proportion of their weight each season?). Secondly, is the rate of change across weeks constant within a season? Finally, does whether the animal was on water regulation the previous week impact the weight loss in the subsequent week? To address this, we first considered the change in weight that occurred from each week (week *i*) to the next (week *i+1*) (using only the body weight measurements from the first day on water regulation, i.e. Monday mornings), expressing this as a % change in weight relative to bodyweight measured in week *i*. We then fitted GLMs to these % values, using three predictors: (1) the animal’s mean weight, (2) the number of weeks since seasonal transition (i.e. since a change in day length), and (3) whether the animal had been on water regulation the previous week (week *i-1*). Data for summer and winter were modelled separately. All single, two and three parameter models (and their interactions) were tested, and in each case only a single parameter contributed significantly to the model fit.

When modelling weight changes in the winter the only parameter to predict changes in the rate of weight gain was the number of weeks since transition. In this model the intercept was significant (beta = 1.38, Table 1), suggesting that typically animals gained 1.38% of their body weight weekly in winter, and the week was also a significant predictor (beta = -0.065, Table 1) indicating that animals initially gained weight more rapidly. In summer the only factor to significantly influence weight loss was whether the animal had been on water regulation the previous week. In this model the intercept was significant (beta = -0.73, indicating a typical loss of 0.73%/week, Table 1), with animals who were on regulation the previous week losing slightly less weight than those who had had access to free water in the previous week (beta = 0.82, Table 1).

**Table 1:** Final models for estimating the % change in body weight from week *i* to *i+1*. The number of weeks since transition, the animals’ mean body weight and whether the animal had been water regulated in week *i-1* were considered as factors (both in single parameter models and full models including interaction terms), with only the number of weeks since transition being predictive for the winter data, and whether the animal had been on water regulation or not the previous week influencing the summer weight data.

**Winter**			
	***Beta***	***t-statistic***	***p-value***
**Intercept**	1.3778	6.3158	6.63E-10
**# weeks since transition**	-0.065161	-3.3247	9.60E-04
**Summer**			
	***Beta***	***t-statistic***	***p-value***
**Intercept**	-0.73	-9.8469	1.65E-21
**Free water previous week**	0.82439	4.1028	4.56E-05

### Animals show repeatable patterns of weight change across multiple years

**Fig 4:**
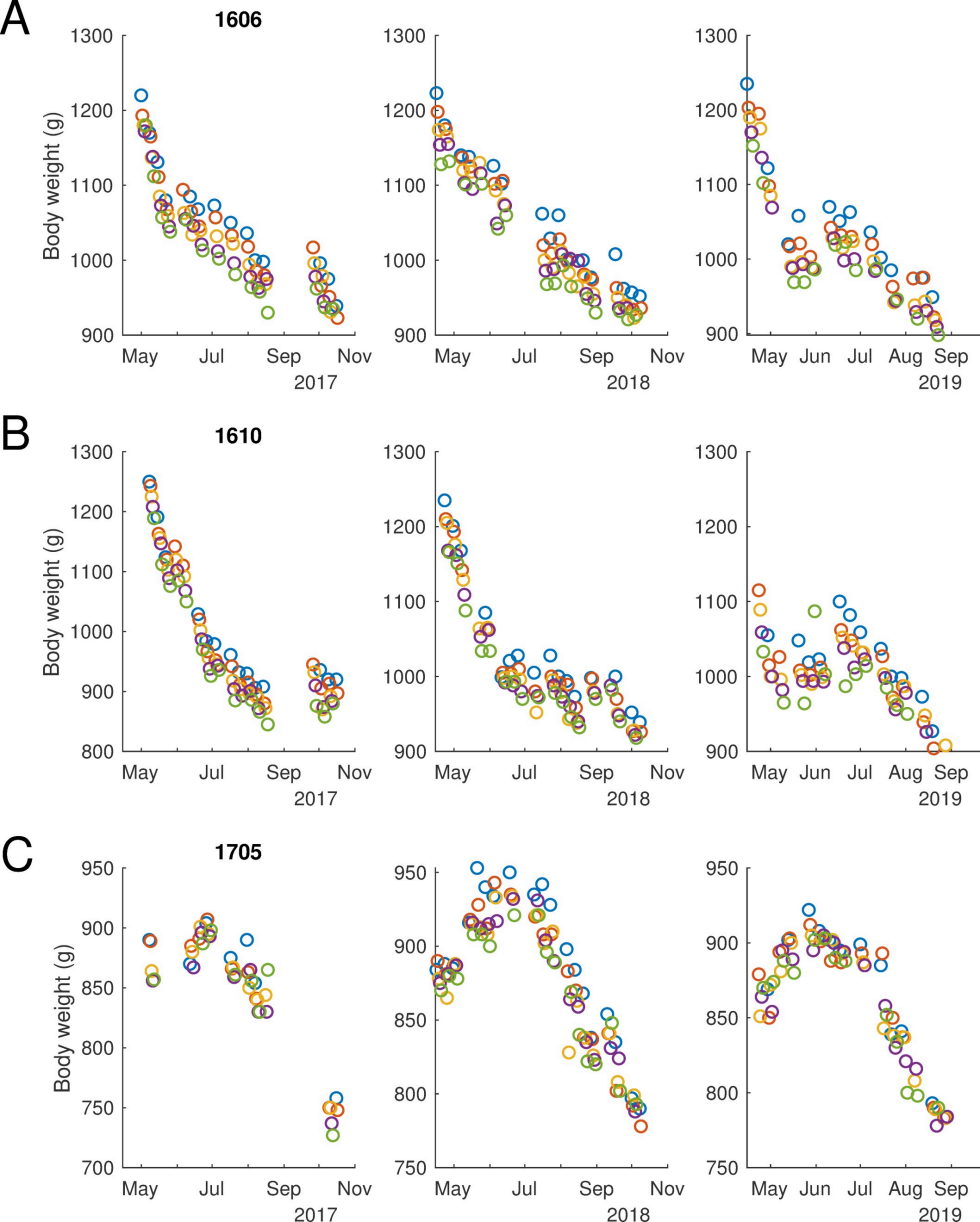
Individuals have distinct but reproduceable weight changes across seasons. Example weight data from three ferrets measured over three consecutive years. Data points recorded are colour coded by day (Monday–Friday: Blue, orange, yellow, purple, green). A, F1606, shows sustained weight loss throughout the summer. B, F1610, shows initial rapid weight loss which stabilises. C, F1705, continues to gain weight after the light change, before beginning to lose weight for the remainder of the season. Patterns A and B were most common, although the pattern evident in C was seen in 5/39 animals.

Despite being able to describe and quantify general trends in our population of animals, we were not able to derive a statistical model that enabled us to successfully predict changes in weight for individual animals (data not shown). Examination of the raw data make it apparent that individual animals follow stereotyped patterns of weight change that were repeatable from year to year, but that this resulted in considerable variability across animals. This is illustrated with three animals, each of which we tracked over three summers, in Fig 4. Some animals showed relatively constant weight loss through the summer (Fig 4A), whereas others showed a more rapid initial loss, before plateauing (Fig 4B). A minority of animals showed a different pattern, continuing to gain weight after the light change, reaching a peak nearly two months later, before experiencing the more typical summer weight loss (Fig 4C, note also the across animal differences in the impact of water regulation visible by comparing blue (Monday) and green (Friday) data points). Together these data illustrate the benefit in obtaining and maintaining long-term weight records for individual animals.

**Fig 5:**
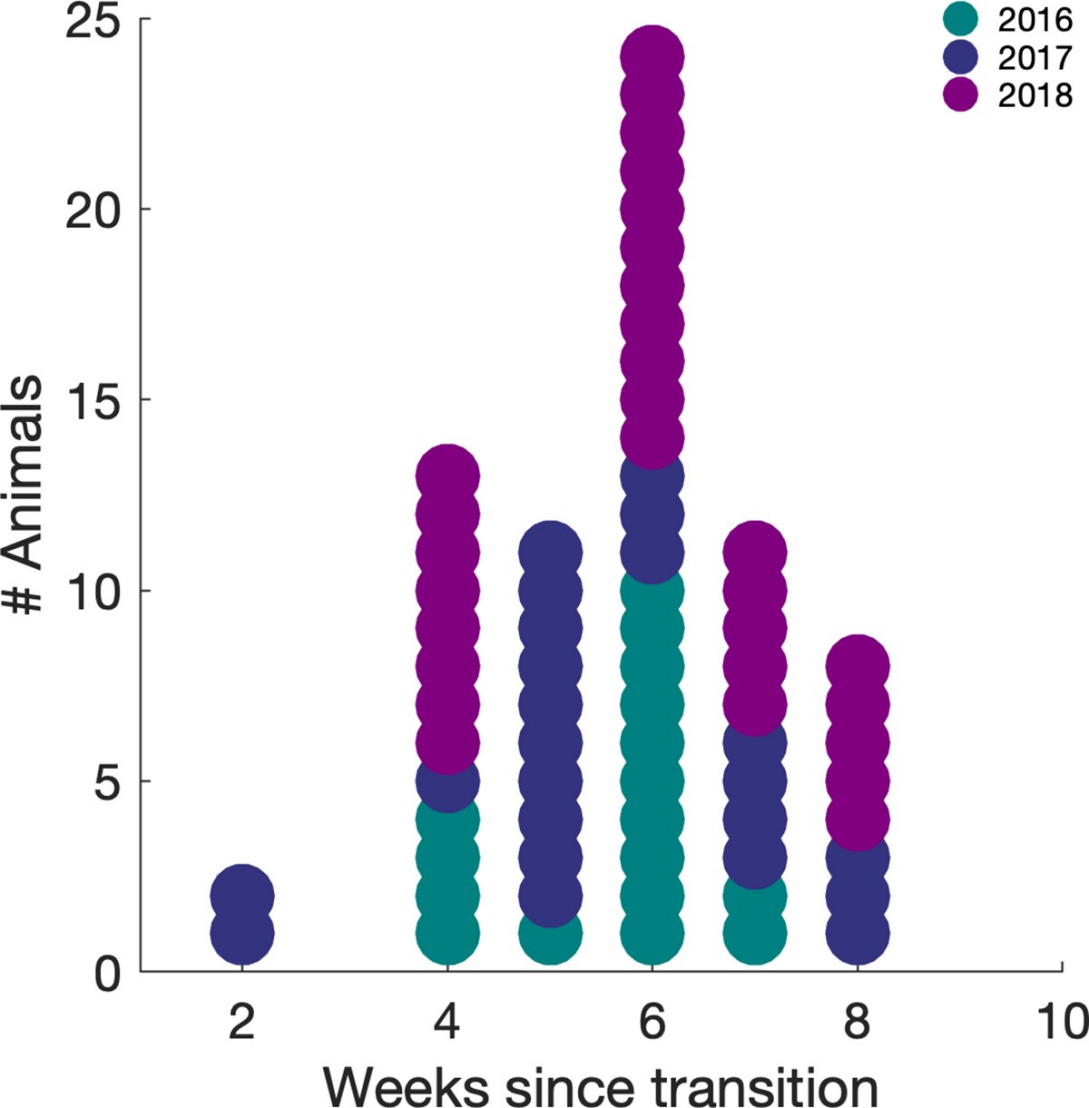
Timing of oestrus relative to daylight change. Dot histogram with each dot representing the timing of oestrus in a single animal colour coded by the year in which the measurement was made 2016, 2017 and 2018.

In addition to body weight data, we also had the timing of oestrus for each animal (69 measurements, 39 unique animals). Oestrus varied from 2 to 8 weeks after the first change in light cycle length in the spring (Fig 5). Timing did not vary significantly across the three years (Kruskalwallis test, p = 0.54), indicating that oestrus occurred at a reliable interval following the light. The average number of weeks between the first light change in the spring and oestrus was 5.7±1.4 weeks. Unfortunately, the number of missing weight values obtained on the week of light change and the week of oestrus precluded any further statistical analysis of the relationship between weight change and oestrus.

## Discussion

Here we provide a normative weight dataset for the healthy female ferret and demonstrate that ferrets show predictable and stereotypical seasonal fluctuations in weight, with most animals gaining around 0.89% of their average body weight per week in winter and losing around 0.65% of their weight per week in summer. Water regulation also causes highly stereotyped changes in body weight which are superimposed upon these seasonal fluctuations.

The cumulative effect of these weekly changes imposed large fluctuations in body weight with animals typically being roughly 15% heavier in winter, and 15% lighter in summer (i.e. a variation of as much as 30% of their mean weight). This pattern of seasonal weight change demonstrated by our ferrets follows previously observed changes [23]. The range of mean weights that we observed across our population were in keeping with previously reported data [21,31,35]. There are many possible physiological contributing factors to this seasonal weight loss including age [31], coat changes [23,27], fat storage, hormone levels such as melatonin [27] and activity levels, which in wild animals are critical for survival [36].

Water regulation imposed an additional pattern of weight changes on animals; weight was lost over the week in both summer and winter. On average, between Monday morning (when water bottles had been removed the previous evening) and Friday morning animals lost around 3% of their body weight in winter, and 4% in summer. Water consumption varies with diet and body mass. Typical daily volumes reported for a ferret can be up to 100ml/day [31, reported for male ferrets which with a typical weight range of 1.2–2.2Kg would give a range of 45-83ml/Kg] and we ensured ferrets received 60ml/kg of water each day of water restriction (which is the amount that animals maintained on laboratory ferret diet, with free access to water, are observed to consume per day). Since the key contributor to weight loss in water regulated animals is thought to be reluctance to eat dry food (rather than dehydration per se) providing animals with water combined with ground pellet diet to form a mash [8] appears to be successful at ensuring weight loss does not exceed more than a few percent. Food and water restriction are common methods used as motivation to train many laboratory animals including ferrets, non-human primates, rats and mice in tasks for research [37], and weight loss is a key marker of health status. Understanding how seasonal fluctuations interact with these effects is therefore important to refine health assessment and ensure the highest standards of animal welfare.

In addition to body weight data, we also had the timing of oestrus for each animal (69 measurements, 39 unique animals). Oestrus varied from 2 to 8 weeks after the first change in light cycle length in the spring with an average value of 5.7±1.4 weeks. Previous research has observed that the cycle of changes in light duration were responsible for initiating oestrus and weight changes but whether there is a causal relationship between the two remains unknown [21]. Further research is required to directly assess whether it is the daylight change itself, weight changes induced by daylight change, or an interaction between the two factors, as in hamsters [38], that induce oestrus.

In other species, research has outlined a possible mechanism driving the photoperiodic responses. Energy balance is regulated through a process where darkness duration induces neuroendocrine activity of melatonin, further mediated by changes in gene expression within the hypothalamus to adjust the energy homeostasis accordingly [39,40]. These effects result in seasonal weight variation and oestrus stimulation, but ferret-specific data is required to determine if they occur through similar mechanisms.

The animals used in this study were all classed as young to middle aged [31] with the oldest animal being 4 years old at the end of the study. Ferret weight also varies across the lifespan, which is on average between 6–8 years [31]. At birth, female ferrets weigh 6-12g and grow rapidly to 550-700g at 10–12 weeks and 600-950g at approximately 16 weeks (i.e. adulthood) [8,21,24,31]. Ferrets are defined as old after the age of 3–4 years and are at greater risk of geriatric diseases including insulinomas and hyperadrenocorticism but also natural weight loss that may be exacerbated during seasonal changes [31,41].

We do not have sufficient repeated data from older animals to determine how ageing interacts with seasonal weight changes. Greater weight loss is observed in aged ferrets (>4 years old) during, and in recovery from, illness [42]. With weight loss an indicator of possible disease, accounting for the age of the animal is important for contextual assessment of health status, and so further research is required to quantify weight changes during ageing and precautionary close observation of weight loss in older animals would be justified.

Pulling together our findings we would expect that animals should gain weight in winter, with an initial increase of around roughly 1.4%/week, declining to 0.7%/week after 10 weeks and 0.1%/week after 20 weeks. In contrast, across the population, the expected weight loss in the summer was roughly linear, with animals losing roughly 0.7%/week, except in weeks which were preceded by access to free water in which weight loss was around 1.5%. However, these statistical relations mask substantial inter-animal variability (as seen in Fig 4) and suggest that if possible, patterns of weight loss/gain should be interpreted in the context of an animal’s own historical data. Superimposed upon these seasonal changes were within-week changes reflecting the fluctuations in body weight that result from fluid regulation. These changes were typically of the order of 3–4% from a Monday morning to Friday morning. Together, these data therefore establish some normative benchmarks for seasonal weight variation in female ferrets that can be incorporated along with other indicators of well-being into the assessment of an animal’s overall condition.
